# Metastatic tracheal melanoma misdiagnosed as chronic obstructive pulmonary disease: A case report

**DOI:** 10.1016/j.rmcr.2025.102262

**Published:** 2025-07-09

**Authors:** Arshdeep Singh Marwaha, Donald Cockcroft, Julian Tam, Brianne Philipenko

**Affiliations:** a Internal Medicine, University of Saskatchewan, Canada; b Division of Respirology and Sleep Medicine, Department of Medicine, University of Saskatchewan, Canada

## Abstract

**Introduction/objective(s):**

Metastatic tracheal melanoma is rare, with fewer than 20 reported cases. This case describes a 62-year-old female with a history of cutaneous melanoma excised 10 years prior, initially misdiagnosed with severe COPD. We highlight the diagnostic challenges when rare metastases mimic common conditions.

**Description:**

Diagnosed with COPD based on dyspnoea and spirometry, the patient later developed worsening symptoms, including haemoptysis, requiring hospitalisation. A chest radiograph was unremarkable, but CT pulmonary angiogram revealed a 1.6 × 1.3 cm tracheal mass. Bronchoscopy confirmed 80–90 % luminal stenosis due to a friable mass, which biopsy identified as tracheal melanoma (BRAF V600E positive). She underwent tumor debulking via rigid bronchoscopy, followed by radiation therapy and vemurafenib.

**Discussion:**

This case represents the longest interval between cutaneous melanoma and tracheal metastasis. Spirometry showed a COPD-like scooping pattern rather than the expected large airway obstruction, delaying diagnosis. New-onset severe airflow obstruction in patients with minimal smoking history should prompt alternative considerations. Advanced imaging and bronchoscopy are essential for early detection. Treatment includes surgical debulking, radiation, and targeted therapy, with follow-up showing symptom resolution and normalised spirometry.

**Conclusion:**

Metastatic tracheal melanoma can mimic COPD, leading to misdiagnosis. The prolonged latency highlights the need for vigilance in melanoma follow-up. Rare airway lesions should be considered in atypical COPD presentations, reinforcing the importance of advanced diagnostic tools for timely identification and treatment.

## Highlights

• Unusual Metastatic Interval: Tracheal melanoma diagnosed 10 years after primary cutaneous melanoma—the longest reported interval to tracheal metastasis.
• COPD Misdiagnosis: Spirometry mimicked small airway obstruction, masking the presence of a central airway tumour.
• Importance of Advanced Imaging and Direct Visualization: CT and bronchoscopy should be considered in atypical or treatment-resistant obstructive lung disease.
• Figures.

## 1 Case presentation

**Fig. 1:**
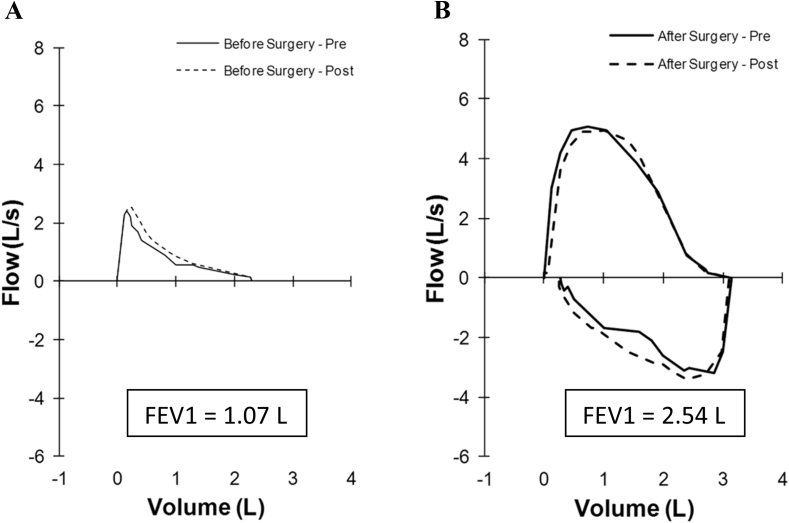
Flow-volume curves. (A) Airflow limitation on initial spirometry. (B) Normalization of expiratory flow rates. 2 months after tumour debulking.

A 62-year-old female was diagnosed with severe chronic obstructive pulmonary disease (COPD), in the primary care setting, due to a five-year history of worsening dyspnoea, and spirometry demonstrating severe airflow obstruction with a forced expired volume in 1 s (FEV_1_) of 1.07 L (38 %), forced vital capacity (FVC) 2.52 L (73 %), and FEV1/FVC (0.43) (Fig. 1A). Her past medical history was significant for cutaneous melanoma, excised from her back 10 years prior, and a remote 5-pack-year history of smoking. She subsequently presented to her community hospital with dyspnoea, productive cough, and several days of hemoptysis. She was transferred to a tertiary care hospital, where she was treated for an acute exacerbation of COPD. Her blood pressure was 113/64, heart rate of 117 beats per minute, respiratory rate of 20 breaths per minute, and oxygen saturation of 95 %, with 3 L/min of supplemental oxygen. Coarse breath sounds with probable upper airway inspiratory noise were noted.

**Fig. 2:**
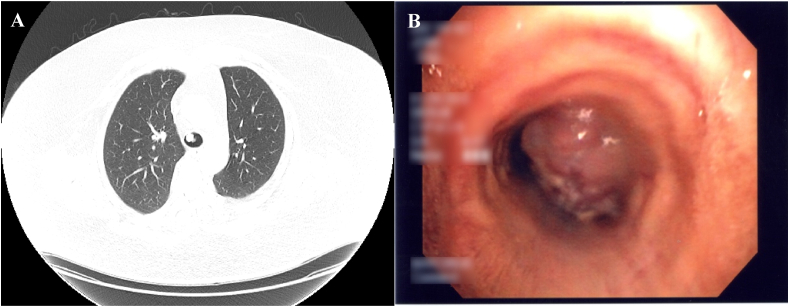
(A) Axial computed tomography of the thorax with lung windows demonstrates an intraluminal anterior tracheal mass. (B) Tracheal mass visualized on flexible bronchoscopy.

**Fig. 3:**
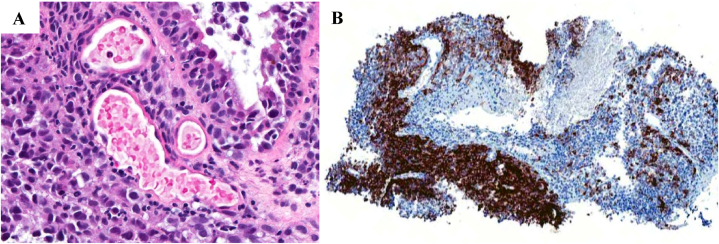
(A) Hematoxylin and Eosin (H&E) staining demonstrates an infiltrative neoplasm composed of pleomorphic, hyperchromatic cells with prominent nucleoli and high mitotic activity. The tumor cells invade the submucosa and are associated with a lymphocytic infiltrate. (B) Immunohistochemical staining with HMB-45 shows strong, patchy cytoplasmic positivity in tumor cells, confirming melanocytic origin and supporting the diagnosis of metastatic melanoma.

The chest radiograph was unremarkable, without hyperinflation, masses, or vascular deficiency. Due to concern of a possible pulmonary embolism, a contrast-enhanced computted tomography pulmonary angiogram was undertaken. While negative for pulmonary embolism, it revealed a 1.6 × 1.3 cm mass on the anterior tracheal wall, immediately above the level of the carina (Fig. 2A). Flexible bronchoscopy was undertaken to visualize this mass directly. The bronchoscopy identified a friable tracheal mass causing 80–90 % luminal stenosis (Fig. 2B). Histopathological analysis of the biopsy confirmed tracheal melanoma, with positivity for the BRAF V600E mutation (Fig. 3A and B, respectively).

She was referred to the Thoracic Surgery service, where tumour debulking via rigid bronchoscopy was undertaken. Following surgery, she underwent radiation therapy and treatment with vemurafenib. Follow-up after 12 months revealed clinical stability. Two months after surgery, the patient had complete resolution of dyspnoea, cough, and hemoptysis. Expiratory flow rates had normalised with an FEV1 of 2.53 L (92 %), FVC 3.11 L (91 %), and FEV1/FVC 0.82 (Fig. 1B).

## 2 Discussion

**Fig. 4:**
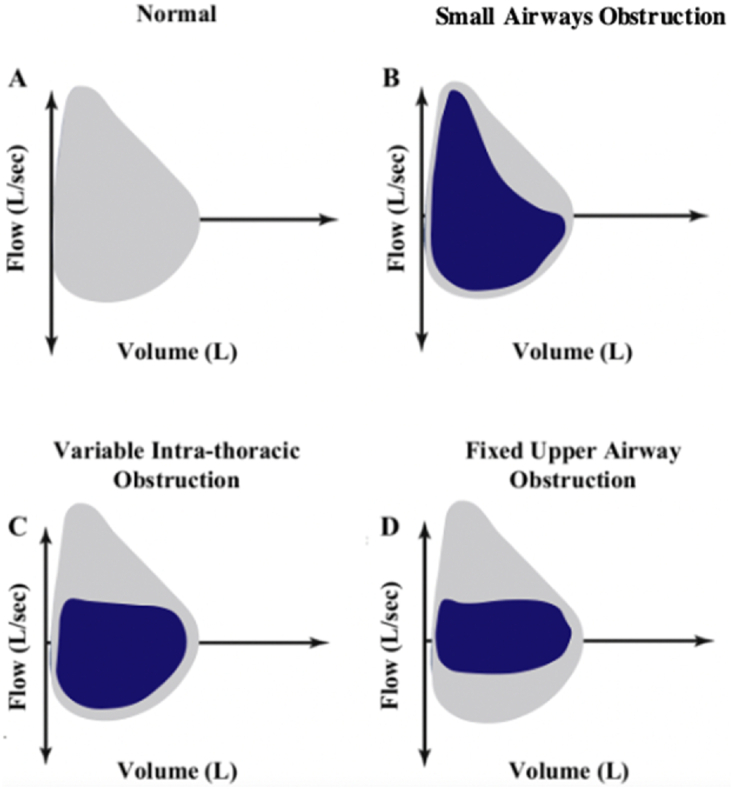
Typical Flow-volume curve illustrations of (A) Normal Airflow (B) Small Airways Obstruction (i.e. COPD) (C) Variable Intra-thoracic obstruction (D) Fixed Upper Airway Obstruction.

Flow-volume loops illustrate lung function and can be used to assess and manage lung diseases such as COPD. As seen in asthma and COPD, the flow-volume loop for small airway obstruction is characterised by a concave or “scooped out” appearance of the expiratory loop, indicating reduced expiratory flow and prolonged expiration due to diffuse small airway obstruction. Inspiratory flow may remain relatively normal, although it can be slightly reduced depending on the severity of the disease. Variable extra-thoracic large airway obstruction is characterized by airway collapse on inspiration. On a flow volume loop, this would appear as the flattening of the inspiratory loop. By contrast, variable intra-thoracic large airway obstruction is characterized by airway collapse on expiration. On a flow volume loop, this would appear as the flattening of the expiratory loop. Lastly, fixed large airway obstruction leads to flattening of both inspiratory and expiratory loops. Our patient with tracheal melanoma exhibited a flow-volume loop characterised by scooping during expiration, typical of the diffuse small airway obstruction seen in COPD (Fig. 4B), leading to an initial misdiagnosis of COPD. Typically, a lesion in the trachea would manifest as either a variable pattern of intra-thoracic large airway obstruction (Fig. 4C) or fixed large airway obstruction (Fig. 4D). However, the absence of these expected large airway patterns contributed to overseeing an endotracheal lesion at that stage. An endotracheal tumour mimicking COPD has previously been reported [6]. Spirometry obtained prior to presentation confirmed the presence of significant expiratory airflow obstruction. As such, she was inappropriately treated as a person with COPD for several years. Concurrent inspiratory flows were not obtained due to significant dyspnoea and were not clinically relevant in this context, given that the tumour was not extra-thoracic.

Metastatic tracheal melanoma is a rare malignancy occurring in only 5 % of patients with extrapulmonary endobronchial metastases [1,2]. A literature review revealed that fewer than 20 English language case reports had been published. While relatively rare, primary tracheal melanoma is somewhat more common [3]. The possibility of primary tracheal melanoma was considered unlikely in our patient due to the history of a previously excised cutaneous melanoma 10 years prior [4]. The reported duration between the diagnoses of cutaneous melanoma and tracheal metastasis in the literature ranges from two months to eight years [5,6]. Our patient's delay of ten years following treatment of cutaneous melanoma to the diagnosis of tracheal metastasis is the longest reported to date.

A retrospective analysis of patients with primary tracheal tumours reported that cough, hemoptysis, and dyspnoea were the most common symptoms among individuals with malignant lesions [7]. Spirometry and flow-volume curves commonly reveal physiologically variable intrathoracic large airway obstruction or, less likely, fixed large airway obstruction [8,9]. As demonstrated in our case, a tracheal mass may be occult on chest radiography but is more readily detected via chest computed tomography or direct visualization through laryngoscopy or bronchoscopy.

## 3 Conclusion

Metastatic tracheal melanoma is a rare diagnosis. While previous case reports have discussed this entity, to our knowledge, this case represents the longest time period between primary tumour and evidence of distant metastasis. While airflow obstruction mimicking COPD caused by tracheal melanoma has been demonstrated in a previous case report [6], this case reinforces the need to consider the natural history of diagnoses. The initial diagnosis in this case was COPD, in the context of a minimal, remote smoking history. The sudden development of severe airflow obstruction in a previous healthy patient without a history of atopy or previous asthma should call this diagnosis into question.

## CRediT authorship contribution statement

**Arshdeep Singh Marwaha:** Writing – review & editing, Writing – original draft, Visualization, Formal analysis. **Donald Cockcroft:** Writing – review & editing, Supervision. **Julian Tam:** Writing – review & editing, Writing – original draft, Supervision. **Brianne Philipenko:** Writing – review & editing, Supervision.

## Funding

None.

## Declaration of competing interest

The authors declare that they have no known competing financial interests or personal relationships that could have appeared to influence the work reported in this paper.
